# Clinical evaluation of periodontal pathogen levels by real-time polymerase chain reaction in peri-implantitis patients

**DOI:** 10.1186/s40729-021-00385-0

**Published:** 2021-10-06

**Authors:** Taichi Ito, Gentaro Mori, Yukari Oda, Tomoki Hirano, Hodaka Sasaki, Shinya Honma, Yoshitaka Furuya, Yasutomo Yajima

**Affiliations:** Department of Oral and Maxillo-Facial Implantology, Tokyo Dental College, 1-2-2 Masago, Mihama-ku, Chiba, 261-8502 Japan

**Keywords:** Clinical research, Diagnosis, Microbiology, Peri-implantitis

## Abstract

**Objective:**

The mechanisms underlying the onset and progression of peri-implantitis are similar to those of periodontitis, and the causative bacteria are believed to similar. Previous studies support an association between peri-implantitis and periodontal pathogen. Thus, we investigated the bacterial flora of peri-implantitis patients in comparison to those of healthy implant and periodontitis patients.

**Materials and methods:**

In total, 70 patients visiting Tokyo Dental College Chiba Hospital were divided into four groups: healthy, periodontitis, healthy implant, and peri-implantitis. For each group, the following five periodontal pathogens were detected using real-time polymerase chain reaction: *Porphyromonas gingivalis*, *Aggregatibacter actinomycetemcomitans*, *Tannerella forsythia*, *Treponema denticola*, and *Prevotella intermedia*.

**Results:**

The average copy number of total bacteria was significantly higher in the periodontitis group than in the other groups. *P. gingivalis* was detected in the periodontitis and peri-implantitis groups at levels as high as 18.92% and 12.29%, respectively, and *P. intermedia* was found in the peri-implantitis group at a rate of 2.06%. Nevertheless, periodontal pathogens were generally detected at lower levels in the peri-implantitis group than in the periodontitis group.

**Conclusion:**

We found lower bacterial counts in the peri-implantitis group relative to the periodontitis group. Our results suggest that the peri-implant tissue is less resistant to bacteria, so even a small number of bacteria can be a risk factor for peri-implantitis and the causative agent of peri-implantitis can be bacteria other than periodontal pathogen.

## Introduction

Peri-implantitis is an inflammatory disease that affects the gums and bone structure around an osseointegrated dental implant. Without treatment, it can lead to bone destruction and may interfere with the long-term stability of the implant. The incidence and prevalence of peri-implantitis in dental implant patients has been reported to range from 1 to 56% [1]. Thus, peri-implantitis is a potential risk for any implant patient and it should, therefore, be a major focus of clinical dentistry research.

The mechanisms underlying the onset and progression of peri-implantitis are similar to those of periodontitis. Although many factors can contribute to implant failure, evidence suggests that bacterial infection of peri-implant tissue is a major cause. The causative bacteria are also thought to be similar to periodontitis [2, 3]. Although peri-implantitis and periodontitis are typically caused by bacterial infections, microbiological tests that can be easily applied clinically are yet to be established. Thus, both diseases are often treated without identifying the causative bacteria. In recent years, various chair-side microbial tests have been developed to detect periodontal disease, including enzyme assays, DNA probe methods, and polymerase chain reaction (PCR). None of these tests, however, is widely employed due to their lack of convenience and prohibitive costs [4–7]. However, it is now possible to quantify periodontal pathogens by real-time polymerase chain reaction (RT-PCR) methods, so clinical bacterial tests are being performed more frequently [8–11].

In the present study, we used RT-PCR to investigate the periodontal pathogen flora in patients with peri-implantitis and periodontitis, as well as healthy implant patients, with the aim of assessing the similarity of the periodontal pathogens in each disease.

## Materials and methods

### Patients and clinical evaluation

In total, 70 adult Japanese patients (26 male and 44 female; mean age ± SD = 58 ± 8 years) requesting dental implant treatment and peri-implantitis treatment, who were visiting the Department of Maxillo-facial and Oral Implantology at Tokyo Dental College Chiba Hospital, were evaluated in this study. The health status and background of each patient was recorded, including their age, sex, and probing pocket depth. The patients were also categorized into one of four groups: the healthy, healthy implant, periodontitis, and peri-implantitis groups (Table 1). All probing was carried out with a Williams probe and recorded at six sites (mesiofacial, midfacial, distofacial, mesiolingual, midlingual, and distolingual) in each tooth and in implants. None of the patients had received periodontal treatment or antibiotics for at least 6 months prior to participating in this study. Informed consent was obtained from each patient, and the study was conducted with approval from the Ethics Review Board of Tokyo Dental College (Approval No. 182).

**Table 1:** Classification of experimental groups

Classification	Number (male/female)	Age (mean ± SD)	Probing Pocket Depth (PPD) (mean ± SD) (mm)
Healthy (h) group	10 (4/6)	56.2 ± 3.3	3.26 ± 0.46
Periodontitis (p) group	16 (6/10)	58.7 ± 6.1	7.09 ± 1.38
Healthy implant (hi) group	17 (5/12)	54.5 ± 13.2	3.06 ± 0.62
Peri-implantitis (pi) group	27 (11/16)	62.4 ± 9.8	6.89 ± 1.62

Subjects were divided into four groups based on severity of periodontal disease. Indicate the total number of people, the gender split in each group, mean age

### Sampling

**Fig. 1:**
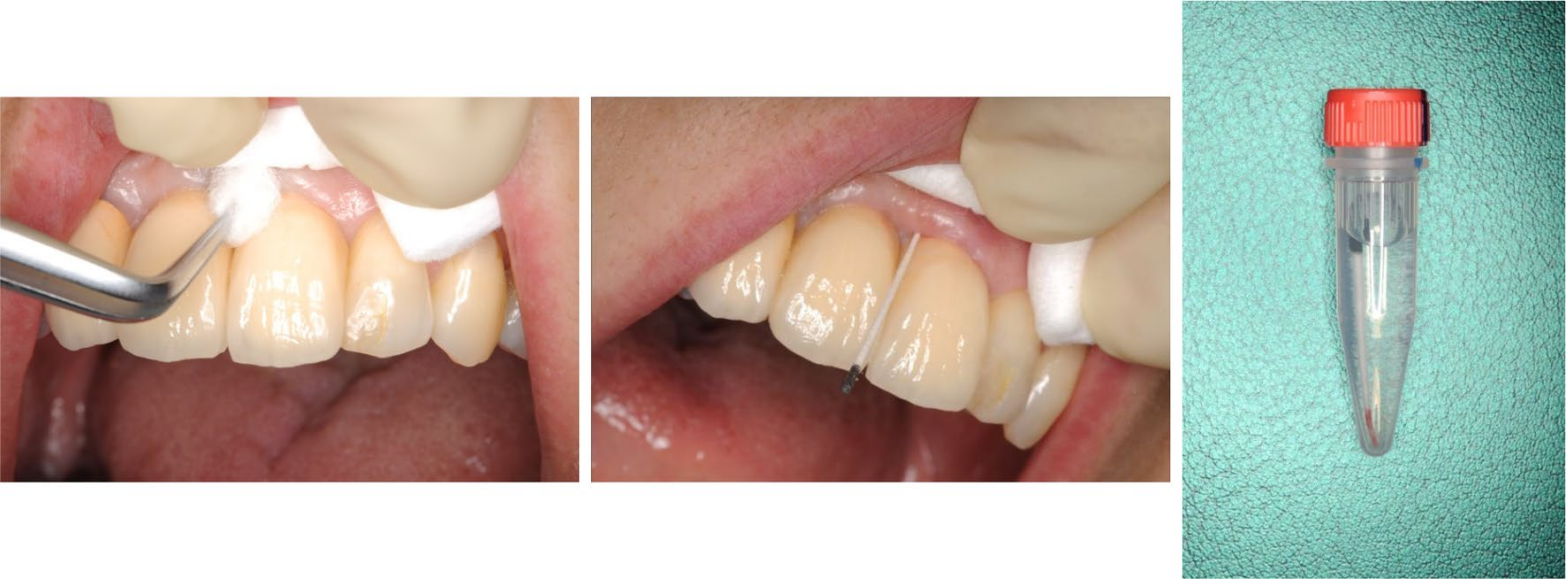
Paper point method for sampling periodontal pathogens

Prior to the sampling described below, the patients had been instructed not to brush their teeth or eat for at least 1 h. At least one deep probing pocket depth site was selected in each patient. Each sampling site was isolated with cotton rolls, and supra-gingival plaque and saliva were carefully removed with sterile cotton pellets before being allowed to air dry. One paper point was inserted into each selected pocket until firm resistance was felt, and the paper points were kept in place for 30 s (Fig. 1). Once removed, the sample was stored at – 20 °C until it was processed.

### RT-PCR

**Fig. 2:**
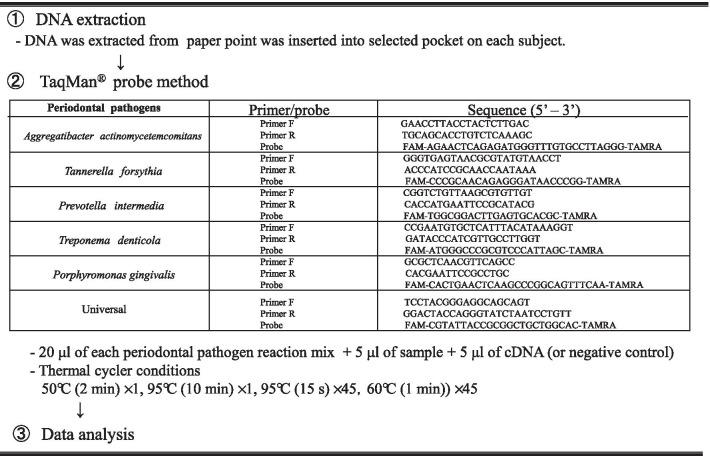
Taqman^®^ real-time polymerase chain reaction assay was employed to determine the count of the targeted infectious agents using the primers and techniques indicated

In preparation for RT-PCR, 100 μl diluted samples were used for automated DNA extraction and purification with a Puregene Core Kit A (Qiagen, Tokyo, Japan). Subsequently, RT-PCR analysis was performed using the TaqMan^®^ probe method (Miroku Medical Laboratory Inc., Nagano, Japan) and the following five periodontopathic bacteria were quantified: *Aggregatibacter actinomycetemcomitans*, *Porphyromonas gingivalis*, *Tannerella forsythia*, *Treponema denticola*, and *Prevotella intermedia*. The primer and probe sets for the five periodontal pathogens targeted, as well as the experimental conditions, are shown in Fig. 2 [12]. Following quantification, the bacterial copy-count numbers and proportion of each bacteria type to total copy-count were determined (with the latter being expressed as a percentage).

### Statistical analysis

SPSS 15.0 for Windows (SPSS Inc., Chicago, IL, USA) was used to conduct statistical analysis. The percentages of each bacteria type to the total copy-count were compared using the Kruskal–Wallis test.* p* values < 0.05 were considered to be statistically significant.

## Results

**Fig. 3:**
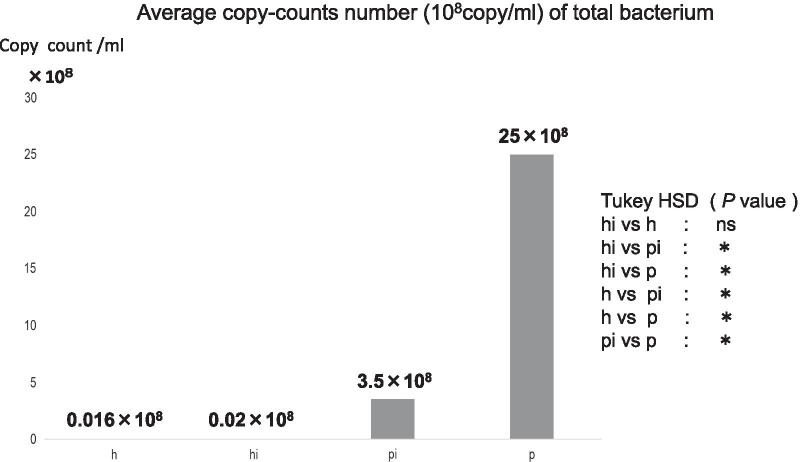
Average copy-count number (10^8^ copies/ml) of the four study groups [healthy (h) group, periodontitis (p) group, healthy implant (hi) group, and peri-implantitis (pi) group]

**Fig. 4:**
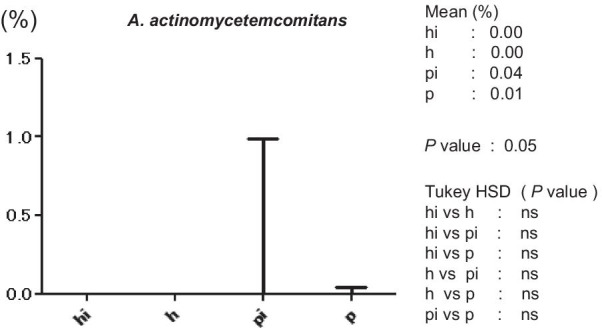
Average proportion (%) of bacteria to total copy-count for *Aggregatibacter actinomycetemcomitans*

**Fig. 5:**
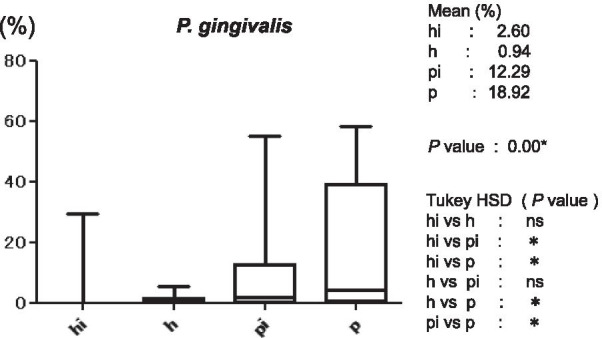
Average proportion (%) of bacteria to total copy-count for *Porphyromonas gingivalis*

**Fig. 6:**
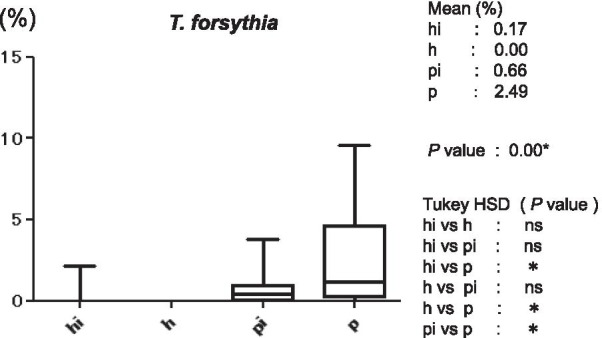
Average proportion (%) of bacteria to total copy-count for *Tannerella forsythia*

**Fig. 7:**
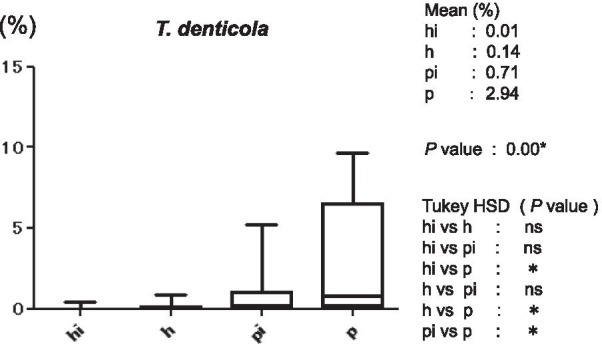
Average proportion (%) of bacteria to total copy-count for *Treponema denticola*

**Fig. 8:**
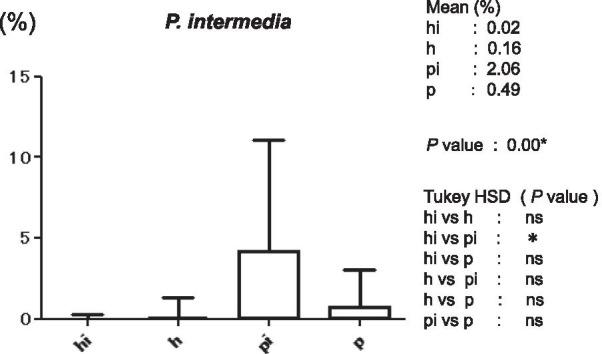
Average proportion (%) of bacteria to total copy-count for *Prevotella intermedia*

Figure 3 shows the average copy-count numbers of total bacteria in each group. The high values were observed in the periodontitis (25 × 10^8^ copy-count/ml) and peri-implantitis (3.5 × 10^8^ copy-count/ml) groups; the copy-count for the periodontitis group was significantly higher than that of any other group. No significant difference was detected in *A. actinomycetemcomitans* levels among the four groups (Fig. 4). However, the average percentage of *P. gingivalis* (relative to total copy-count) was markedly high in the periodontitis and peri-implantitis group (18.92% and 12.29%, respectively), and levels in the periodontitis group were significantly higher than in any other group (*p* < 0.01) (Fig. 5). The average percentages of *T. forsythia* (*p* < 0.01) and *T. denticola* (*p* < 0.01) were also significantly higher in the periodontitis group than in other groups (Figs. 6 and 7). In contrast, the average percentage of *P. intermedia* was markedly high in the peri-implantitis group at 2.07%; indeed, it was significantly higher in the peri-implantitis group than in the healthy implant group (*p* < 0.05) (Fig. 8).

## Discussion

A new finding in this study was that the total number of copies of bacteria comparing the four groups tended to be lower in the peri-implantitis group than in the periodontitis group. However, the periodontal probing depth was the same in the periodontitis and peri-implantitis groups, suggesting that the peri-implant tissues have low resistance to bacteria even if the relative number of bacteria is small; thus, bacteria are clearly a risk factor for the development and progression of peri-implantitis. This finding is supported by reports that experimental periodontitis and peri-implantitis were caused by the formation of supragingival plaque in Beagle dogs [13, 14], and that inflammation of the tissue around the implant increased compared with tissue around natural teeth only 3 months after plaque formation. This suggests that the protective function of the tissue around the implant is inferior to that of the tissue around natural teeth. It has also been suggested that occlusal parafunction as a non-bacterial factor may be the cause of peri-implantitis [15]. However, it has been reported that occlusal parafunction does not affect changes in bone level in the peri-implant tissue [16, 17]. At present, there seems to be no scientific evidence that occlusal parafunctions can be a factor in implant failure.

The results of this study showed that periodontal pathogens were detected more often in peri-implantitis patients than in patients with healthy implants, and that *P. intermedia* was present at significantly higher levels in the peri-implantitis group. This result is consistent with several previous studies of the periodontal pathogens associated with peri-implantitis; indeed, there is a bacteriological link between infection with periodontal pathogens and the failure of implant treatments [18]. In peri-implantitis, the bacterial infection occurs in the peri-implant tissue at the site of osseointegration; this complication can hinder the long-term stability of the implant [19, 20].

Periodontal disease patients are reported to be particularly susceptible to peri-implantitis. *P. gingivalis*, *T. forsythia*, and *T. denticola*, a group of bacteria known as the red complex, are considered to be highly likely to cause periodontal disease [21]. Similarly, these bacteria are linked to peri-implantitis. Maximo et al. reported a high proportion of red complex bacteria in subgingival plaques in peri-implantitis-affected areas [22]. Furthermore, peri-implantitis and periodontal disease-related bacteria have been reported to correlate with the bacteria associated with periodontitis [18]. In the present study, the five bacterial species, including the red complex, *A. actinomycetemcomitans,* and *P. intermedia*, were assessed as to whether they were associated with peri-implantitis and periodontitis. Only *A. actinomycetemcomitans* was rarely detected in patients with these diseases. This may be because *A. actinomycetemcomitans* is typically associated with aggressive periodontitis, which generally affects younger people, whereas the patients with implants in our study were relatively old (mean age 53.3 ± 9.0 years). The similarity in the bacteria detected in peri-implantitis and periodontitis is unsurprising, because peri-implantitis is thought to be caused by the periodontal pathogens transmitted from the periodontal pocket in the same oral cavity and by the same mechanism as periodontitis.

The detection of periodontal pathogen in the peri-implantitis group was lower than that in the periodontitis group, it is possible that bacteria other than periodontal pathogens cause the onset and progression of peri-implantitis. Previous studies have reported that various types of bacteria from chronic periodontitis are detected in affected areas of peri-implantitis [23]. Leonhardt et al. found that while periodontopathic bacteria were detected in about 60% of patients with peri-implantitis, *Staphylococcus* spp., enterobacteria, and *Candida* spp. were also detected in 55% of patients [24]. In studies that comprehensively analyzed the bacterial flora in the area affected by peri-implantitis, *Parvimonas micra*, *Eubacterium* spp., *Peptococcus* spp., and *Butyrivibrio* spp., among other bacteria, were also detected in addition to periodontopathic bacteria [25–27]. Thus, a variety of bacteria are active in peri-implantitis-affected areas; new methods, including next-generation sequencing, will be used in future to search for and identify these bacteria.

It is important that we understand the risk of peri-implantitis and perform periodontal disease-related bacterial tests in patients with peri-implantitis to consider appropriate treatments for this disease. The RT-PCR method used in this study is a highly reliable test method that is rapidly becoming widespread in clinical practice as a test for COVID-19 [28, 29]. At present, the purpose of periodontal pathogen testing is to identify the causative agents of periodontal disease, to screen for high-risk patients with periodontal disease, to acquire information used to inform the patient and choose treatment, and to build toward a scientific consensus on the selection of antibacterial agents. Hereafter, further research aimed at identifying other bacterial species by new bacterial test methods such as the 16S rRNA gene sequence will be needed. The results of the present study suggest that periodontal pathogen tests can be considered for use as effective pre-treatment risk tests and peri-implantitis tests during dental implant treatment.

## Conclusion

The results of this study revealed that the number of bacteria in the peri-implantitis group was lower than that in the periodontitis group. The low resistance of peri-implant tissue to bacteria suggests that even low bacterial counts may also be associated with peri-implantitis. In addition, the detection of periodontal pathogen in the peri-implantitis group was lower than that in the periodontitis group, suggesting that the causative bacteria of peri-implantitis may be bacteria other than periodontal pathogen.

## Abbreviations

RT-PCR: Real-time polymerase chain reaction
*A. actinomycetemcomitans*: *Aggregatibacter actinomycetemcomitans*
*P. gingivalis*: *Porphyromonas gingivalis*
*T. forsythia*: *Tannerella forsythia*
*T. denticola*: *Treponema denticola*
*P. intermedia*: *Prevotella intermedia*
